# Calcium ionophore pre-treatment induces type-1 polarized DCs with enhanced T cell stimulatory function and IL-12 production

**DOI:** 10.1186/2051-1426-1-S1-P199

**Published:** 2013-11-07

**Authors:** Erik Berk, Shuwen Xu, Min Xu, Julia Terhune, Brian Czerniecki

**Affiliations:** 1 Department of Surgery and the Harrison Surgical Research Center, University of Pennsylvania, Philadelphia, PA, USA; 2 Rena Rowan Breast Cancer Center, University of Pennsylvania, Philadelphia, PA, USA

## 

The effective treatment of cancer by immunotherapy requires the induction of high numbers of tumor-specific type-1 polarized T cells. Since DCs are the key antigen-presenting cells capable of activating and polarizing T cells, the generation of type-1 polarized DCs for DC-based anti-cancer therapies is desired. We have previously shown that DCs matured with calcium ionophore (CI) alone results in the generation of type-2 polarized DCs, which lack IL-12 production. However, we now show that pre-treatment of monocyte-derived DCs with CI followed by the maturation with the inflammatory cytokine IFNγ and the TLR agonists LPS (TLR2) and R848 (TLR7/8), results in an enhanced IL-12 production by the DCs compared to DCs matured without CI pre-treatment. The effect of CI on IL-12 production appears to be dependent on the kinetics of CI pre-treatment as well as on the maturation factors used following CI addition. The CI-matured DCs show a more mature phenotype (based on CD83 and CCR7 expression), express higher levels of the co-stimulatory molecule CD70, and have reduced expression of the inhibitory molecule PD-L1 compared to DCs matured without CI-pre-treatment. When loaded with antigen these CI-matured DCs strongly induce T cell proliferation and the expression of the cytolytic proteins granzyme B and perforin by CD8+ T cells in a mixed leukocyte reaction. The ability of CI to induce type-1 polarization with enhanced IL-12 secretion and T cell stimulatory function allows for the development of type-1 polarized DC-based anti-cancer vaccines.

